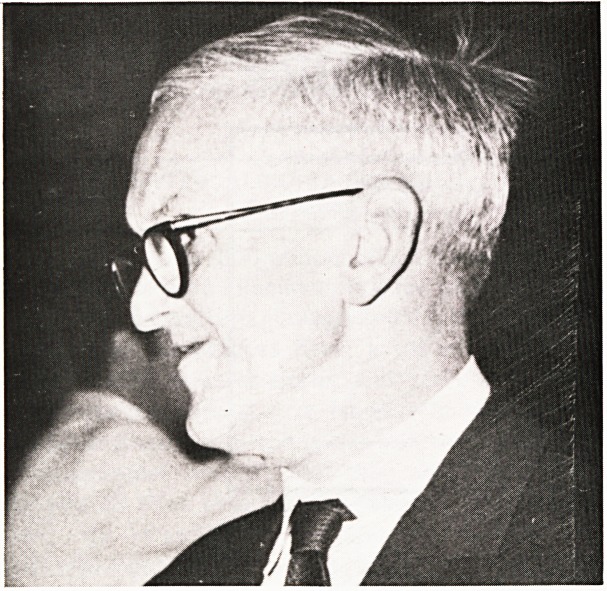# Dr. R. I. S. Gordon

**Published:** 1979

**Authors:** 


### Obituary

**Dr. I. R. S. Gordon, M.D., F.R.C.P., F.R.C.R.**

Dr. Ian Gordon died suddenly on 12th June 1979, at the age of 64. Following two heart attacks in the previous 16 years he had lived a full professional, family and social life until his untimely end.

He graduated M.B., B.Chir. in 1939 from Cambridge and the London Hospital, obtained the M.R.C.P. in 1942 and M.D. in 1944. He passed the D.M.R.D. in 1952 and was awarded the F.R.C.R. without examination in 1975.

Being unfit to serve during the war he worked as medical registrar in Bath, his home city, throughout the war. In 1950, he came to Bristol to train as a radiologist. He spent the rest of his working life in Bristol and became one of the most significant radiologists in this country in a rapidly developing field of the discipline. Within the Teaching Hospital Group in the University of Bristol, he took over responsibility for Radiology at the Children's Hospital and the Maternity Hospital. In time he became one of the leading paediatric radiologists not only in this country, but also in Europe. He was a founder member of the European Paediatric Radiologists Club and never missed its annual meeting. He was a founder member of the Radiologists Group within the British Paediatric Association which held its first meeting very successfully in 1979 in York.

In Bristol he was highly respected and admired, and held in great affection. He was at his best as a teacher. He had the gift of stimulating younger people to think. He always had time to stop what he was doing and to explain. He communicated well and easily and understandably at face-to-face level. The book on 'Diagnostic Radiology in Paediatrics', which he published three years ago jointly with Dr. F. G. M. Ross, is already the standard book on paediatric radiology in this country. He had been for many years secretary of the medical staff committee of the Children's Hospital and later, its Chairman. He held innumerable regular weekly and fortnightly consultation sessions with paediatricians and surgeons and others, which were enormously popular as teaching/learning sessions with both undergraduate and postgraduate students. The work of the Bristol Children's Hospital literally revolved round the Radiology Department with him as its central pivot. However, he never overlooked his responsibility to the whole Department and took an active part in all its regular teaching sessions, always contributing cases to the weekly Tuesday lunch-time case discussion seminars. Similarly for 25 years he organised the weekly academic lecture that takes place during University term-time. He routinely attended and took part in the monthly meeting of the Division and for a decade before such things as divisions were thought of he acted as secretary of the departmental committee. He was largely instrumental in the creation of the Bristol Bone Dysplasia Registry and cases were sent to him from far and wide for opinion. He had been its secretary and film curator since its inception.

He was a quiet unassuming man. He and Diana, his wife, were hospitable and many of the overseas trainee radiologists who came to Bristol for their training remember the quiet warmth and genuine welcome given to them in their country home, where their six children had grown up, and where grandchildren visit not infrequently. They were intrepid travellers and caravanners, driving to many remote parts of Europe for holidays and even on one occasion using their caravan as base, while he attended the meeting of the European Paediatric Radiology Association. We were fortunate to have him as a colleague and we honour his memory.

## Figures and Tables

**Figure f1:**